# 固相萃取-超高效液相色谱-串联质谱法同时测定水中61种激素

**DOI:** 10.3724/SP.J.1123.2023.11014

**Published:** 2024-09-08

**Authors:** Yueqin CHEN, Ming MA, Hongdan XU, Chunyan PAN

**Affiliations:** 1.江苏联合职业技术学院无锡卫生分院,江苏无锡 214000; 1. Wuxi Health Branch, Jiangsu United Vocational and Technical College, Wuxi 214000, China; 2.无锡市疾病预防控制中心,江苏无锡 214023; 2. Wuxi Center for Disease Control and Prevention, Wuxi 214023, China

**Keywords:** 超高效液相色谱-串联质谱, 固相萃取, 激素, 水, ultra performance liquid chromatography-tandem mass spectrometry (UPLC-MS/MS), solid phase extraction (SPE), hormone, water

## Abstract

甾体激素类的污染及危害问题不容忽视,为了实现更加全面、准确的高通量分析,研究建立了固相萃取-超高效液相色谱-串联质谱同时测定水中糖皮质激素类(48种)、盐皮质激素类(1种)、雄激素类(4种)及孕激素类(8种)等共61种激素成分的多残留分析方法。采用HC-C18固相萃取柱对大体积(1 L)水样中的目标化合物进行富集净化,乙腈洗脱,以BEH C18色谱柱分离,以0.1%甲酸水溶液和乙腈为流动相梯度洗脱,用超高效液相色谱-串联质谱仪分离和检测。质谱采用电喷雾正离子电离、多反应监测模式,外标法定量。61种激素在相应的范围内,线性关系良好,相关系数均大于0.99,方法检出限为0.05~1.50 ng/L,在低、中、高3个加标水平下的回收率为62.3%~125.2%,相对标准偏差为1.1%~10.5%。将建立的方法应用于太湖流域的地表水、相关区域的地下水以及末梢水的分析,部分地表水和地下水中检出了可的松、丙酸氟替卡松、环索奈德、倍他米松双丙酸酯、氯倍他松丁酸酯、戊酸双氟可龙、卤倍他索丙酸酯、异氟泼尼龙、二氟孕甾丁酯和己酸羟孕酮等10种成分,其余51种成分未检出。该方法操作简单,灵敏度高,准确度好,对后续水安全的监测以及溯源调查有重要意义。对水中激素水平进行了地区分析,提出了未来污水处理工艺应将激素残留作为目标物进行针对性处理的建议。

## Abstract (en)

Concerns over the emergence of steroid hormones as pollutants in water have grown. Steroid hormone compounds present challenges in the simultaneous detection of total residual hormones owing to their analogous structures and diverse types. In this study, we established a rapid and high-throughput continuous online method based on solid phase extraction (SPE) coupled with ultra performance liquid chromatography-tandem mass spectrometry (UPLC-MS/MS) for the simultaneous determination of 61 hormone components, including 48 glucocorticoids, 1 mineralocorticoid, 4 androgens, and 8 progesterones, in water.

Various SPE columns were investigated to assess their extraction efficiency for enriching and purifying target compounds in a large sample volume (1 L). An HC-C18 SPE column was selected because of its superior performance. Acetonitrile was used as a washing solution during SPE to ensure that the majority of the tested substances achieved recoveries exceeding 70% and effectively avoid interferences from water-soluble components.

Various C8 and C18 columns were tested, and the optimal HPLC conditions for hormone retention were established. We systematically evaluated different UPLC columns and mobile phases, including methanol-water and acetonitrile-water systems with 0.1% formic acid added to the aqueous phase. The optimized UPLC conditions were as follows: BEH C_18_ column (100 mm×2.1 mm, 1.7 μm); column temperature, 40 ℃; flow rate, 0.3 mL/min; injection volume, 5 μL; mobile phase A: 0.1% formic acid aqueous phase; mobile phase B: acetonitrile. Gradient elution was performed as follows: 0-0.5 min, 30%B; 0.5-15.0 min, 30%B-75%B; 15.0-18.0 min, 75%B-98%B; 18.0-19.0 min, 98%B; 19.0-19.1 min, 98%B-30%B; 19.1-20.0 min, 30%B.

Both positive- and negative-ion modes were explored in the UPLC-MS/MS experiment to obtain the full scan of the parent ions, and positive mode was finally selected for electrospray ionization (ESI). Two product ions exhibiting strong signals and minimal interference were selected for quantitative and qualitative ion analyses, using an external standard method for quantification. MS/MS was performed in positive-ion (ESI^+^) mode with multiple reaction monitoring (MRM) scanning. The MS/MS parameters were as follows: atomizing gas pressure, 379 kPa; curtain air pressure, 241 kPa; spray voltage, 5500 V; desolvation temperature, 550 ℃; collision exit voltage (CXP), 13 V; intake voltage (EP), 10 V; and residence time of each ion pair, 0.5 ms. Other instrument settings, such as the collision energy and declustering voltage, were also optimized.

The 61 hormones exhibited excellent linear relationships within their corresponding concentration ranges, with correlation coefficients greater than 0.99. The method detection limits (MDLs) were in the range of 0.05-1.50 ng/L. The average recoveries of the 61 hormones across three spiked levels ranged from 62.3% to 125.2%, with relative standard deviations (RSDs, *n*=6) of 1.1%-10.5%.

Using this method, we successfully detected 10 hormone components (cortisone, fluticasone propionate, ciclesonide, betamethasone dipropionate, clobetasone butyrate, diflucortolone valerate, halobetasol propionate, isoflupredone, difluprednate, and hydroxyprogesterone caproate) in various surface water and groundwater samples collected from the Taihu Basin region.

The SPE-UPLC-MS/MS method presented in this paper is simple, highly sensitivity, and exceptionally accurate. Thus, it exhibits promising potential for tracing targeted hormone residues in water and will be of great value in monitoring and ensuring water safety. Finally, a regional analysis was conducted on the hormone levels in water, and suggestions were made for the targeted treatment of hormone residues in future sewage treatment processes.

甾体激素类主要包括肾上腺皮质激素和性激素两大类,长期低剂量摄入甾体激素对人体存在一定的健康隐患与风险^[1,2]^。目前,环境领域中对甾体激素类物质的研究较少,而且对水中甾体激素调查研究相关的报道更少。

常用的检测痕量残留激素的分析方法有气相色谱-质谱法^[3⇓-5]^、电化学法^[6]^、酶免疫分析法^[7]^、放射免疫法^[8]^、高效液相色谱法^[9⇓-11]^、液相色谱-三重四极杆质谱法^[12]^等,其中,免疫分析法测定范围较窄,易出现结果假阳性和交叉污染的情况,多用于检测常见、单一性激素。高效液相色谱法检测器灵敏度及准确性存在一定的限制,分析速度较慢。气相色谱-串联质谱法(GC-MS)测定激素成分需要化学衍生,前处理操作繁琐,且目前尚无一种衍生试剂能够对所有激素进行衍生化。超高效液相色谱-三重四极杆质谱(UPLC-MS/MS)因其具有检测灵敏度高、选择性好、特异性好及分析时间短等特点,是目前应用最广泛的激素残留定量分析方法之一^[13,14]^。

本研究建立了固相萃取-UPLC-MS/MS同时测定水中糖皮质激素类(48种)、盐皮质激素类(1种)、雄激素类(4种)及孕激素类(8种)等4类共61种激素成分的分析方法。该法前处理操作简便快捷,全流程有机溶剂用量少,检测激素数量多,测定灵敏度高,准确度好,可为后续水环境中激素的监测和相关健康风险评估提供技术支持,同时对激素溯源调查有一定的参考价值。

## 1 实验部分

### 1.1 仪器、试剂与材料

超高效液相色谱仪-Triple Quad 5500质谱仪(美国Sciex公司); 3-30K离心机(德国Sigma公司); Vortex genie-2涡旋混合器(美国SI公司); Arium pro超纯水发生器(德国Sartorius公司); KS 600D医用超声波清洗机(宁波海曙科生超声设备有限公司); N-EVAPTM111氮吹仪(美国Organomation Associates公司); Multi Reax试管振荡器(德国Heidolph公司); Supelco 12通道半自动反相固相萃取装置;HC-C18固相萃取小柱(上海安谱实验科技股份有限公司);0.45 μm微孔滤膜(天津市津腾实验设备有限公司)。

61种激素标准品(详见表1)购自中国食品药品检定研究院、德国Dr. Ehrenstorfer公司及上海安谱实验科技股份有限公司。

**表1:** 61种激素的保留时间和质谱参数

No.	Compound	CAS No.		*t*/min		Parent ion (*m/z*)		Daughter ions (*m/z*)		Declustering potentials/V	Collison energies/eV
1	triamcinolone (曲安西龙)	124-94-7		2.08		395.2		357.2^*^/225.1		82/82	17/26
2	prednisolone (泼尼松龙)	50-24-8		3.22		361.2		343.2^*^/147.2		85/85	14/34
3	prednisone (泼尼松)	50-03-2		3.25		359.2		341.2^*^/147.2		85/85	15/35
4	isoflupredone (异氟泼尼龙)	338-95-4		3.28		379.2		359.3^*^/341.3		70/70	13/18
5	hydrocortisone (氢化可的松)	50-23-7		3.34		363.2		121.1^*^/327.2		136/136	21/30
6	cortisone (可的松)	50-06-5		3.43		361.3		163.2^*^/121.1		140/140	31/40
7	methylprednisolone (甲基泼尼松龙)	83-43-2		4.16		375.2		339.2^*^/161.2		66/66	14/28
8	dexamethasone (地塞米松)	50-02-2		4.31		393.2		373.4^*^/355.2		78/78	15/15
9	betamethasone (倍他米松)	378-44-9		4.33		393.2		337.3^*^/355.2		81/81	15/19
10	flumethasone (氟米松)	2135-17-3		4.41		411.3		253.2^*^/121.0		80/80	22/50
11	beclomethasone (倍氯米松)	4419-39-0		4.65		409.2		391.2^*^/279.3		84/84	15/29
12	betamethasone 21-acetate (醋酸倍他米松)	987-246-6		4.84		435.3		277.1^*^/171.0		80/80	27/39
13	desonide (地索奈德)	638-94-8		4.85		417.2		147.0^*^/173.0		70/70	39/33
14	triamcinolone diacetate (曲安西龙双醋酸酯)	67-78-7		5.08		479.2		441.2^*^/321.1		81/81	14/19
15	fludroxycortide (氟氢缩松)	1524-88-5		5.21		437.2		361.2^*^/285.2		155/155	24/33
16	prednisolone 21-acetate (醋酸泼尼松龙)	52-21-1		5.24		403.2		147.1^*^/385.2		62/62	35/13
17	fluocortolone (氟可龙)	152-97-6		5.35		377.4		303.3^*^/171.2		66/66	16/26
18	chlormadinone acetate (醋酸氯地孕酮)	302-22-7		5.41		405.1		267.3^*^/309.3		90/90	21/29
19	desoximetasone (去羟米松)	382-67-2		5.45		377.2		147.0^*^/171.0		70/70	28/29
20	fludrocortisone 21-acetate (醋酸氟氢可的松)	514-36-3		5.45		423.2		239.2^*^/343.2		83/83	34/29
21	prednisone 21-acetate (醋酸泼尼松)	125-10-0		5.65		401.2		147.2^*^/295.2		80/80	23/39
22	deflazacort (地夫可特)	14484-47-0		5.74		442.3		124.1^*^/142.1		81/81	65/45
23	cortisone 21-acetate (醋酸可的松)	50-04-4		5.86		403.2		163.2^*^/343.2		140/140	31/26
24	halometasone (卤美他松)	50629-82-8		5.92		445.3		169.0^*^/155.3		148/148	30/45
25	testosterone (睾酮)	58-22-0		6.18		289.2		109.0^*^/97.1		113/113	29/30
26	methylprednisolone 21-acetate (甲基泼尼松龙醋酸酯)	53-36-1		6.36		417.2		161.1^*^/253.2		85/85	28/28
27	dexamethasone 21-acetate (醋酸地塞米松)	1177-87-3		6.73		435.3		309.3^*^/337.0		50/70	18/17
28	budesonide (布地奈德)	51333-22-3		6.76		431.2		147.1^*^/413.2		83/83	15/42
29	gestrinone (孕三烯酮)	16320-04-0		6.82		309.1		199.2^*^/241.1		120/120	29/43
30	finasteride (非那雄胺)	98319-26-7		6.85		373.3		317.2^*^/305.3		138/138	31/41
31	hydrocortisone 17-butyrate (丁酸氢化可的松)	13609-67-1		6.92		433.3		327.2^*^/345.2		78/78	23/15
32	methyltestosterone (甲基睾酮)	58-18-4		6.92		303.2		97.0^*^/108.9		110/110	30/29
33	fluorometholone 17-acetate (氟米龙醋酸酯)	3801-06-7		7.22		433.3		327.2^*^/345.2		78/78	23/15
34	clobetasol 17-propionate (丙酸氯倍他索)	25122-46-7		7.84		461.3		355.2^*^/373.2		88/88	18/13
35	norgestrel (炔诺孕酮)	797-63-7		7.92		313.2		109.0^*^/245.3		115/115	24/33
36	triamcinolone acetonide 21-acetate (醋酸曲安奈德)	3870-07-3		7.96		477.2		339.2^*^/321.2		80/80	22/23
37	hydrocortisone 17-valerate (氢化可的松戊酸酯)	57524-89-7		8.13		447.3		345.3^*^/121.1		75/75	16/40
38	medrysone (甲羟松)	2668-66-8		8.16		345.2		327.3^*^/135.2		100/100	20/27
39	fluocinonide (氟轻松)	356-12-7		8.21		495.2		337.2^*^/121.1		82/82	24/60
40	diflorasone diacetate (醋酸双氟拉松)	33564-31-7		8.34		495.2		279.2^*^/317.2		85/85	20/23
41	hydrocortisone aceponate (醋丙氢可的松)	74050-20-7		8.46		461.2		309.3^*^/387.2		80/80	15/26
42	difluprednate (二氟孕甾丁酯)	23674-86-4		9.08		509.2		303.1^*^/279.1		80/80	19/21
43	betamethasone 17-valerate (戊酸倍他米松)	2152-44-5		9.12		477.2		355.3^*^/279.3		83/83	18/24
44	methylprednisolone aceponate (醋丙甲泼尼龙)	86401-95-8		9.29		473.3		161.0^*^/381.2		80/80	15/26
45	prednicarbate (泼尼卡酯)	73771-04-7		9.65		489.2		381.3^*^/115.1		80/80	16/25
46	loteprednol etabonate (氯替泼诺)	82034-46-6		9.74		461.3		359.2^*^/265.3		68/68	17/27
47	amcinonide (安西奈德)	51022-69-6		9.89		503.2		339.2^*^/321.2		90/90	24/25
48	alclomethasone dipropionate (阿氯米松双丙酸酯)	66734-13-2		9.95		521.4		301.2^*^/279.2		106/106	22/21
49	halobetasol propionate (卤倍他索丙酸酯)	66852-54-8		9.98		485.4		121.0^*^/276.3		109/109	34/51
50	megestrol acetate (醋酸甲地孕酮)	595-33-5		9.99		385.1		325.1^*^/261.3		90/90	20/26
51	progesterone (孕酮)	57-83-0		10.01		315.2		97.3^*^/109.0		100/100	32/26
52	tixocortol pivalate (新戊酸替可的松)	55560-96-8		10.05		463.2		361.2^*^/343.2		120/120	25/26
53	fluticasone propionate (丙酸氟替卡松)	80474-14-2		10.35		501.2		313.2^*^/293.2		84/84	22/20
54	hydrocortisone 21-acetate (醋酸氢化可的松)	50-03-3		10.38		405.3		309.2^*^/326.9		105/105	24/22
55	medroxyprogesterone 17-acetate (醋酸甲羟孕酮)	71-58-9		10.44		387.2		122.9^*^/327.0		91/91	19/33
56	betamethasone dipropionate (倍他米松双丙酸酯)	5593-20-4		10.68		505.3		411.2^*^/319.2		82/82	15/21
57	clobetasol 17-butyrate (氯倍他松丁酸酯)	25122-57-0		11.81		479.3		279.2^*^/343.2		95/95	19/22
58	diflucortolone valerate (戊酸双氟可龙)	59198-70-8		11.83		479.4		355.3^*^/85.1		110/110	21/31
59	testosterone propionate (丙酸睾酮)	57-85-2		13.65		345.3		109.2^*^/97.1		140/140	36/31
60	hydroxyprogesterone caproate(己酸羟孕酮)	630-56-8		14.37		429.1		313.2^*^/271.3		76/76	20/27
61	ciclesonide (环索奈德)	126544-47-6		15.94		541.3		323.2^*^/173.2		81/81	25/36

*Quantitative ion.

乙腈、甲醇、乙酸乙酯、叔丁基甲醚、丙酮、正己烷(色谱纯,德国Merck公司);氯化钠、无水硫酸镁(分析纯,国药集团化学试剂有限公司); PSA、C18、GCB(分析纯,上海安谱实验科技股份有限公司);实验用水为实验室自制超纯水。

### 1.2 实验条件

#### 1.2.1 色谱条件

色谱柱:BEH C18柱(100 mm×2.1 mm, 1.7 μm,美国Waters公司),柱温:40 ℃,流速:0.3 mL/min;进样量:5 μL;流动相:A为0.1%甲酸水溶液,B为乙腈;梯度洗脱程序:0~0.5 min, 30%B; 0.5~15.0 min, 30%B~75%B; 15.0~18.0 min, 75%B~98%B; 18.0~19.0 min, 98%B; 19.0~19.1 min, 98%B~30%B; 19.1~20.0 min, 30%B。

#### 1.2.2 质谱条件

电喷雾电离源,正离子模式;扫描方式:多反应监测(MRM)模式;雾化气压力:379 kPa;气帘气压力:241 kPa;喷雾电压:5500 V;去溶剂温度:550 ℃;碰撞出口电压(CXP): 13 V;摄入电压(EP): 10 V;每个离子对驻留时间:0.5 ms;每种化合物的检测离子对、去簇电压(DP)、碰撞能量(CE)等质谱参数见表1。

### 1.3 标准溶液配制

标准品用甲醇配制成100 μg/mL的单标准储备液,于-20 ℃冰箱保存。临用前用甲醇配制成1 μg/mL的混合标准溶液,再用甲醇稀释,配制成所需浓度。

### 1.4 样品采集与保存

样品采集于棕色玻璃瓶中,采集水样前,先用水样洗涤取样瓶及塞子3次,充满后至溢出,盖上塞子,于4 ℃保存,及时预处理,保存时间一般不超过7天。

### 1.5 样品前处理

取水样1 L,经0.45 μm微孔滤膜过滤,依次用6 mL甲醇和6 mL水进行HC-C18固相萃取柱活化平衡后,水样以1 mL/min的速度流过HC-C18萃取柱,水样加载完毕后,用6 mL超纯水对萃取柱进行淋洗,继续抽真空10 min,使固相萃取柱干燥。用6 mL乙腈对萃取柱进行洗脱,洗脱液收集在10 mL玻璃离心管中,40 ℃水浴下,用氮气吹至近干,加入0.1%甲酸水溶液-乙腈(7∶3, v/v)溶解残留样品,至1 mL,经0.22 μm尼龙滤膜过滤后待测。

## 2 结果与讨论

### 2.1 实验条件考察

#### 2.1.1 质谱分析条件的优化

使用流动注射方式分析目标化合物的单标准溶液,分别在正、负离子模式下进行母离子全扫描,得到各化合物的准分子离子。因待测目标化合物均在正离子模式下易电离,响应更好,所以在正离子模式下选择响应值高的离子作为母离子,然后对母离子进行二级扫描,获得相应的离子碎片,选择信号强、干扰小的两个子离子分别作为定量、定性离子,最后优化碰撞能量及碰撞电压,使每个目标待测物的特征离子对强度达到最优,优化后的质谱条件见表1。

#### 2.1.2 色谱条件的优化

大部分待测分析物属于弱极性且相对分子质量较大的化合物,在C18、C8固定相中均有较好保留。实验比较了不同色谱柱(BEH C18(100 mm×2.1 mm, 1.7 μm)、BEH C8(100 mm×2.1 mm, 1.7 μm)、Eclipse plus C18(100 mm×2.1 mm, 1.8 μm,)、Eclipse plus C8(100 mm×2.1 mm, 1.8 μm)和Kinetex-C18(100 mm×2.1 mm, 1.7 μm))的分离效果。结果显示,各待测物在C18柱上的保留行为、峰形及分离效果更好,其中在BEH C18色谱柱上的分离度与灵敏度最高,故选择该色谱柱进行后续实验。

实验首先比较了水-甲醇体系及水-乙腈体系作为流动相时61种激素的色谱响应,发现61种激素在水-甲醇(见图1a)体系下的整体响应不如水-乙腈体系(见图1b),且出峰时间整体偏后并较为集中,因此初步确定采用水-乙腈体系作为流动相。

**图1:**
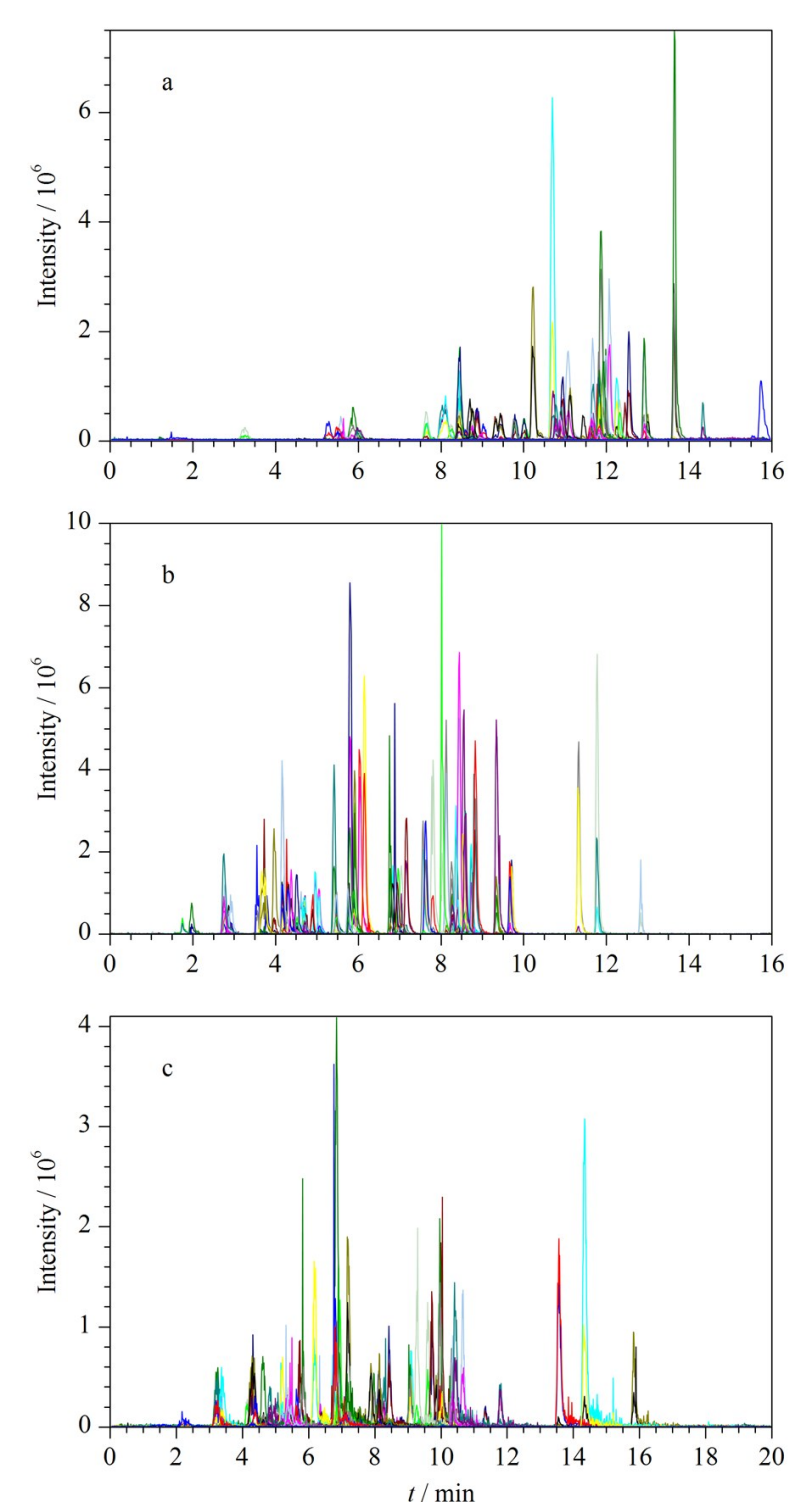
61种激素在不同流动相下的总离子流色谱图

随后实验比较了水-乙腈及0.1%甲酸水溶液-乙腈分别作为流动相时61种激素的峰形和响应。结果显示,流动相中存在0.1%甲酸后,峰形及分离效果有所改善(见图1c)。

#### 2.1.3 固相萃取条件的选择

为提高前处理效果,保证分析的准确度和灵敏度,在目标化合物的萃取过程中,对洗脱剂和SPE小柱进行了优化。

结合前期已有研究^[15]^,再根据待测物极性,分别选用丙酮、乙腈、叔丁基甲醚、乙酸乙酯、甲醇等试剂作为洗脱剂,以标准品的回收率为评价指标。结果发现,采用乙腈作为洗脱剂,既能减少待测组分的损失,保证绝大部分待测物的回收率在70%以上,又能避免部分水溶性成分的干扰。

随后实验考察了HC-C18、Oasis HLB、Oasis MAX、Oasis MCX和Oasis WAX等5种固相萃取小柱对50 ng/mL混合标准溶液的萃取效果(见图2),结果显示使用HC-C18小柱后,待测物的回收率在70%以上。

**图2:**
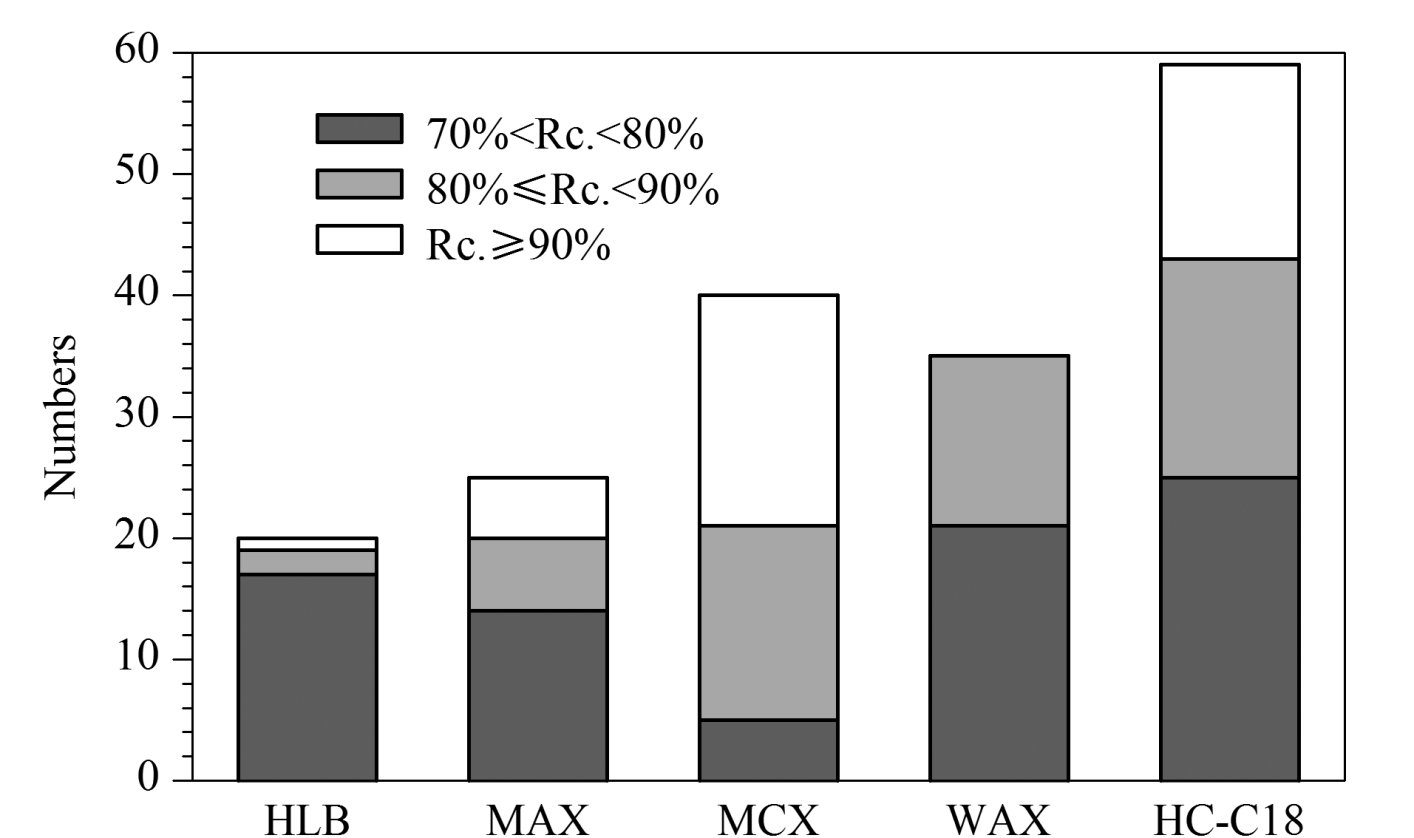
采用不同类型固相萃取小柱时61种激素的回收率分布

最终采用HC-C18固相萃取小柱,乙腈作为洗脱剂。

#### 2.1.4 水样pH值的影响

实验所采集的水样pH值均在6.5~8.5范围内。在未调节水样pH的情况下,61种激素的回收率均能在70%以上。因此实验无需刻意调节水样的pH值。

### 2.2 方法学验证

#### 2.2.1 线性范围、检出限及定量限

配制61种激素的系列混合标准溶液,以各分析物的质量浓度为横坐标,各激素定量离子对的峰面积为纵坐标绘制标准曲线,得到的线性回归方程见表2,线性相关系数(*r*)均大于0.99,在线性范围内线性良好。通过向水样品中添加61种激素,分别以各目标物色谱峰的3倍和10倍信噪比(*S/N*)来确定检出限和定量限,结合所取样品体积及最终定容体积,计算得出水样中61种目标物的方法检出限(MDL)和方法定量限(MQL),分别为0.05~1.50 ng/L和0.15~4.50 ng/L(见表2)。

**表2:** 61种激素的回归方程、线性范围、相关系数、方法检出限、方法定量限、回收率和相对标准偏差(*n*=6)

No.		Compound	Regression equation	Linear range/ (ng/mL)		*r*		MDL/ (ng/L)		MQL/ (ng/L)		Recovery/ %		RSD/ %
1		triamcinolone	*y*=1.18×10^4^*x*-2.57×10^3^	0.10-100		0.9990		0.05		0.15		81.7-89.5		2.7-5.2
2		prednisolone	*y*=5.61×10^4^*x*-2.51×10^4^	0.10-100		0.9949		0.05		0.15		90.3-92.4		3.1-4.6
3		prednisone	*y*=4.43×10^4^*x*+7.56×10^4^	0.50-100		0.9989		0.30		1.00		82.8-88.6		3.5-5.4
4		isoflupredone	*y*=2.11×10^4^*x*-9.12×10^3^	0.20-100		0.9986		0.15		0.50		79.3-85.1		2.8-6.5
5		hydrocortisone	*y*=1.46×10^4^*x*+8.47×10^3^	0.50-100		0.9985		0.30		1.00		89.0-92.3		2.5-7.4
6		cortisone	*y*=4.46×10^4^*x*-1.22×10^3^	0.50-100		0.9979		0.30		1.00		85.4-92.4		6.5-8.7
7		methylprednisolone	*y*=1.94×10^4^*x*+3.53×10^4^	0.50-100		0.9941		0.30		0.10		83.2-125.2		1.1-6.8
8		dexamethasone	*y*=6.24×10^4^*x*-2.28×10^3^	0.50-100		0.9998		0.30		1.00		79.1-95.3		4.5-7.1
9		betamethasone	*y*=3.78×10^4^*x*+1.67×10^4^	0.10-100		0.9992		0.06		0.20		84.9-93.7		5.9-8.5
10		flumethasone	*y*=1.83×10^4^*x*+5.72×10^2^	0.20-100		0.9988		0.15		0.50		83.4-85.4		2.8-6.7
11		beclomethasone	*y*=4.82×10^4^*x*+8.67×10^2^	0.50-100		0.9996		0.30		1.00		73.7-80.4		3.8-9.1
12		betamethasone 21-acetate	*y*=3.31×10^3^*x*+1.88×10^3^	1.00-100		0.9988		1.00		3.00		75.2-87.0		4.9-8.3
13		desonide	*y*=2.65×10^4^*x*+7.99×10^3^	0.20-100		0.9996		0.15		0.50		80.6-88.7		5.8-7.3
14		triamcinolone diacetate	*y*=9.29×10^3^*x*+1.99×10^3^	0.20-100		0.9983		0.15		0.50		89.-106.9		4.9-6.8
15		fludroxycortide	*y*=1.22×10^4^*x*-7.05×10^3^	0.20-100		0.9989		0.15		0.50		62.3-85.3		3.2-6.4
16		prednisolone 21-acetate	*y*=4.34×10^4^*x*-2.50×10^3^	0.20-100		0.9980		0.15		0.50		91.5-107.1		2.2-6.4
17		fluocortolone	*y*=1.99×10^4^*x*+9.60×10^2^	0.20-100		0.9968		0.15		0.50		87.9-94.7		5.4-6.5
18		chlormadinone acetate	*y*=1.77×10^4^*x*+2.89×10^3^	0.50-100		0.9997		0.50		1.50		75.8-100.8		4.2-8.4
19		desoximetasone	*y*=1.55×10^4^*x*-1.13×10^4^	0.50-100		0.9991		0.50		1.50		83.1-107.6		4.1-5.9
20		fludrocortisone 21-acetate	*y*=1.07×10^4^*x*-8.44×10^3^	0.20-100		0.9978		0.20		0.60		85.5-94.8		3.2-5.5
21		prednisone 21-acetate	*y*=1.25×10^4^*x*+5.32×10^3^	0.50-100		0.9987		0.50		1.50		78.3-88.1		6.2-7.5
22		deflazacort	*y*=4.13×10^4^*x*+1.24×10^4^	0.50-100		0.9996		0.50		1.50		65.1-95.1		5.4-9.4
23		cortisone 21-acetate	*y*=1.18×10^4^*x*-2.57×10^3^	0.20-100		0.9991		0.15		0.50		86.7-92.4		4.7-6.4
24		halometasone	*y*=4.15×10^3^*x*+1.42×10^3^	0.20-100		0.9992		0.20		0.60		86.9-93.6		5.6-9.1
25		testosterone	*y*=5.42×10^4^*x*+2.72×10^4^	0.50-100		0.9997		0.50		1.50		95.7-108.4		6.2-7.8
26		methylprednisolone 21-acetate	*y*=1.18×10^4^*x*-2.66×10^2^	0.20-100		0.9990		0.15		0.50		88.5-100.7		1.7-3.5
27		dexamethasone 21-acetate	*y*=1.40×10^4^*x*+2.97×10^3^	0.50-100		0.9992		0.50		1.50		79.4-95.6		5.2-7.5
28		budesonide	*y*=4.12×10^4^*x*+2.11×10^4^	0.50-100		0.9998		0.50		1.50		87.7-122.1		3.8-6.7
29		gestrinone	*y*=9.32×10^4^*x*-2.03×10^4^	0.20-100		0.9999		0.15		0.50		94.6-101.3		4.1-7.3
30		finasteride	*y*=2.49×10^5^*x*+1.41×105^3^	0.10-100		0.9990		0.05		0.20		89.1-109.9		4.5-6.9
31		hydrocortisone 17-butyrate	*y*=1.83×10^4^*x*-1.27×10^1^	0.50-100		0.9999		0.50		1.50		78.5-110.0		4.9-7.5
32		methyltestosterone	*y*=7.77×10^4^*x*+2.27×10^4^	0.10-100		0.9998		0.10		0.30		81.2-103.8		6.1-9.3
33		fluorometholone 17-acetate	*y*=1.24×10^5^*x*+5.77×10^4^	0.20-100		0.9992		0.15		0.50		95.1-109.0		4.2-8.4
34		clobetasol 17-propionate	*y*=3.78×10^4^*x*+3.00×10^4^	0.10-100		0.9988		0.05		0.20		88.5-98.7		2.8-4.9
35		norgestrel	*y*=4.10×10^4^*x*-7.97×10^3^	0.50-100		0.9996		0.50		1.50		78.6-115.9		6.2-9.5
36		triamcinolone acetonide 21-acetate	*y*=3.19×10^4^*x*+1.97×10^4^	0.20-100		0.9990		0.15		0.50		79.7-91.1		3.5-8.6
37		hydrocortisone 17-valerate	*y*=3.68×10^4^*x*-4.99×10^3^	0.20-100		0.9997		0.15		0.50		94.1-104.4		5.2-8.4
38		medrysone	*y*=2.38×10^4^*x*-1.40×10^4^	0.50-100		0.9981		0.50		1.50		85.7-96.5		2.5-5.3
39		fluocinonide	*y*=2.57×10^4^*x*+1.43×10^4^	0.50-100		0.9997		0.50		1.50		85.4-94.2		3.1-6.5
40		diflorasone diacetate	*y*=3.20×10^4^*x*-1.10×10^4^	0.50-100		0.9996		0.30		1.00		86.6-88.6		2.7-7.8
41		hydrocortisone aceponate	*y*=4.54×10^4^*x*-1.28×10^4^	0.20-100		0.9993		0.15		0.50		92.6-96.7		2.5-5.4
42		difluprednate	*y*=4.07×10^4^*x*-5.91×10^3^	0.20-100		0.9998		0.15		0.50		80.9-92.5		4.6-6.8
43		betamethasone 17-valerate	*y*=5.17×10^4^*x*-1.68×10^3^	0.10-100		0.9986		0.10		0.30		79.2-91.7		3.4-8.9
44		methylprednisolone aceponate	*y*=8.95×10^4^*x*+3.27×10^4^	0.20-100		0.9996		0.20		0.60		73.8-88.6		6.7-9.4
45		prednicarbate	*y*=7.72×10^4^*x*+6.92×10^4^	0.10-100		0.9983		0.10		0.30		87.5-95.1		5.3-7.9
46		loteprednol etabonate	*y*=7.87×10^4^*x*+6.10×10^3^	0.20-100		0.9999		0.20		0.60		93.3-96.4		3.4-5.7
47		amcinonide	*y*=2.56×10^4^*x*+9.28×10^3^	0.50-100		0.9993		0.50		1.50		83.3-94.5		3.5-8.9
48		alclomethasone dipropionate	*y*=2.18×10^4^*x*+6.65×10^3^	0.20-100		0.9996		0.20		0.60		94.4-96.7		2.7-7.5
49		halobetasol Propionate	*y*=9.40×10^3^*x*+1.19×10^3^	0.50-100		0.9995		0.50		1.50		88.9-104.9		4.2-6.6
50		megestrol acetate	*y*=1.16×10^5^*x*+9.63×10^4^	0.20-100		0.9983		0.20		0.60		84.6-100.1		3.8-5.9
51		progesterone	*y*=1.47×10^5^*x*-4.18×10^4^	0.20-100		0.9998		0.20		0.60		95.6-107.3		3.7-6.8
52		tixocortol pivalate	*y*=1.93×10^4^*x*+7.89×10^3^	1.50-100		0.9999		1.50		4.50		94.5-100.8		3.6-8.1
53		fluticasone propionate	*y*=4.68×10^4^*x*-4.99×10^3^	0.20-100		0.9996		0.20		0.60		101.1-106.5		2.1-6.5
54		hydrocortisone 21-acetate	*y*=4.81×10^4^*x*+2.15×10^4^	0.50-100		0.9993		0.50		1.50		85.5-98.6		5.1-7.5
55		medroxyprogesterone 17-acetate	*y*=9.50×10^4^*x*-4.70×10^4^	0.20-100		0.9994		0.20		0.60		101.6-103.4		5.4-9.7
56		betamethasone dipropionate	*y*=8.14×10^4^*x*+6.00×10^4^	0.10-100		0.9981		0.10		0.30		84.6-98.9		2.5-6.9
57		clobetasol 17-butyrate	*y*=3.07×10^4^*x*+2.35×10^3^	0.20-100		0.9998		0.20		0.60		69.3-121.4		2.5-10.5
58		diflucortolone valerate	*y*=2.19×10^4^*x*+1.53×10^4^	0.50-100		0.9992		0.50		1.50		100.5-105.6		2.5-6.5
59		testosterone Propionate	*y*=1.00×10^5^*x*-8.15×10^3^	0.50-100		0.9998		0.50		1.50		91.5-105.3		5.7-9.3
60		hydroxyprogesterone caproate	*y*=7.49×10^4^*x*+4.00×10^4^	0.10-100		0.9997		0.10		0.30		92.5-106.4		6.4-8.5
61		ciclesonide	*y*=5.51×10^4^*x*+3.02×10^3^	0.20-100		0.9996		0.20		0.60		95.4-101.5		3.2-6.9

*y*: peak area; *x*: mass concentration, ng/mL.

#### 2.2.2 准确度和精密度

向实验用水中分别添加适量混合标准溶液(20、50和80 ng/L),按照本文建立的方法进行测定,通过添加回收试验考察方法的准确度和精密度。结果显示,在低、中、高3个加标水平下61种激素的回收率为62.3%~125.2%,相对标准偏差为1.1%~10.5%(见表2)。方法的准确度和精密度符合方法学验证要求。

### 2.3 与其他方法的比较

将固相萃取方法与UPLC-MS/MS相结合,建立了一种可快速测定水中4类61种激素的分析方法,将本文建立的方法与已有方法相比(见表3),本方法检测激素数量多,同时还有较好的回收率和较低的方法检出限。

**表3:** 本方法与其他方法的比较

Ref.	Number of analytes	Matrix	Type	Method	Ion sources	Recovery/%		MDL/(ng/L)	
[16]	12	lake water	4	fully automatic SPE device	ESI^+^/ESI^-^	-		-	
[17]	11	river water	1	DSPME	ESI^+^/ESI^-^	53.6-	90.4	0.2-	10.4
[18]	26	source water	4	SPE	ESI^+^/ESI^-^	51.6-	127	0.01-	3.0
[19]	4	surface water	3	LLE	ESI^+^	72-	141	0.01-	0.20
This method	61	water	4	SPE	ESI^+^	62.3-	125.2	0.05-	1.50

DSPME: dispersed solid phase microextraction.

### 2.4 实际样品测定

#### 2.4.1 水中激素含量情况

2023年2月至4月期间,将建立的方法应用于太湖流域的地表水、相关区域的地下水以及末梢水的分析,部分地表水及地下水样本中分别检出了可的松(5.6~203.6 ng/L)、丙酸氟替卡松(33.6 ng/L)、环索奈德(14.0 ng/L)、倍他米松双丙酸酯(14.8 ng/L)、氯倍他松丁酸酯(40.6 ng/L)、戊酸双氟可龙(37.4 ng/L)、卤倍他索丙酸酯(22.6 ng/L)、异氟泼尼龙(1.0 ng/L)、二氟孕甾丁酯(4.5 ng/L)和己酸羟孕酮(0.8~11.1 ng/L)等10种成分,其余51种激素均未检出。

#### 2.4.2 水中激素水平地区分析

2007年,Chang等^[20]^首次报道了环境中皮质激素的含量水平,其研究表明:在中国城市污水和地表水中存在较高浓度的糖皮质激素,污染水平远高于雌激素。其中皮质醇含量最高,达到120 ng/L,其次是可的松,达到86 ng/L,而2023年采集到的太湖流域水样中,可的松含量最高已达203.6 ng/L。2017年,Bai等^[21]^检测分析了从美国米德湖码头、拉斯维加斯湾和博尔德岛等地采集到的湖水样本和贻贝样本。结果显示,水样中未检测到该实验所测定的5种类固醇激素(17*α*-乙炔基雌二醇、17*β*-雌二醇、雌酮、孕酮、睾酮),仅存在药品和个人护理产品(0.49~36 ng/L),但是睾酮是贻贝组织中含量最高的类固醇(6.3~20 ng/g dw)。2017年,Chiu等^[22]^检测了深圳地区的鱼类和贻贝,从中检测到自然存在的激素以及17*α*-乙炔雌二醇,其总含量高达(315.6±6.5) ng/g dw。2018年的枯水期(1月)和丰水期(7月),林粲源^[23]^对珠三角地区的河流进行了43个采样点的水样采集,24种皮质激素在河水样品中均被检出,检出率为4%(丁酸氯倍他松)~52%(布地奈德),表明它们在珠三角河流中广泛存在,其中有14种皮质激素在枯水期和丰水期均被检出。在珠江三角洲地区的多个污水处理厂的进水中同样检测到多种皮质激素,主要包括皮质醇、可的松和地塞米松^[24⇓-26]^。Durcik等^[27]^在2021年5月至8月间分别采集了斯洛文尼亚相关地区的河流地表水、地下水、处理厂进水和处理厂废水,并进行了25种皮质激素监测指标的检测,有18种成分被检出,包括雌酮、17*β*-雌二醇、睾酮、孕酮、17*α*-乙炔基雌二醇、炔诺酮、左炔诺孕酮、替勃龙、孕二烯、屈螺酮和醋酸氯地孕酮等,处理厂进水和废水中的激素含量是河流水和地下水中激素含量的10倍乃至百倍以上。

随着人类活动范围越来越大,天然激素和人工合成激素会继续被使用并通过环境传播或食物链富集,导致在水环境中能被高频检出,不断地间接或直接影响人体生理代谢过程。

皮质激素在天然水环境和污水处理厂出厂水中已存在,但目前的污水处理工艺还未将该类物质作为主要目标物进行针对性处理,未来的风险评估及技术进步会推进该项处理技术的革新。

## 3 结论

本文建立了水中61种激素的UPLC-MS/MS分析方法,对这61种激素进行了定性定量分析,该方法的线性范围、检出限、回收率及精密度等方法学指标可满足分析要求,且方法简便快速,溶剂用量少,激素检测种类广、数量多,可为水环境中激素的监测和风险评估提供技术支持。
